# Feasibility Exploration of High-Resolution MRI Plaque Features for Assessing Outcomes of Intracranial Angioplasty and Stenting in Ischemic Stroke Patients

**DOI:** 10.31083/RN44261

**Published:** 2025-12-24

**Authors:** Kai Mao, XiangYu Meng, LingYou Chen, Jie Yu, Hao Guo, SiJia Hao, Hui Li, CongHui Li

**Affiliations:** ^1^Department of Neurosurgery, The First Hospital of Hebei Medical University, Hebei Medical University, 050030 Shijiazhuang, Hebei, China

**Keywords:** intracranial atherosclerotic stenosis (ICAS), stroke, radiology, atherosclerotic plaques, high-resolution MRI, estenosis aterosclerótica intracraneal (ICAS, intracranial atherosclerotic stenosis), accidente cerebrovascular, radiología, placas ateroscleróticas, resonancia magnética (RM) de alta resolución

## Abstract

**Objective:**

To evaluate the feasibility of plaque-based radiomics extracted from high-resolution magnetic resonance imaging (HR-MRI) data for assessing the short-term outcomes of endovascular treatment in patients with symptomatic intracranial artery stenosis.

**Methods:**

HR-MRI was performed on patients with symptomatic intracranial artery stenosis. Plaque-based radiomics describing the morphological features and pixel value of the image were extracted from the HR-MRI data. Demographic features were also collected. The short-term favorable outcome was defined by a postoperative residual stenosis rate <35% with the absence of perioperative complications. Univariate analysis was conducted to identify features associated with favorable outcomes. Based on the results of this analysis, a prediction model was developed using logistic regression. The performance of both clinical and radiomic models was evaluated using the receiver operating characteristic (ROC) curve and the area under the curve (AUC).

**Results:**

From January 2022 to December 2023, 42 consecutive patients with symptomatic intracranial artery stenosis were enrolled. Digital subtraction angiography (DSA) revealed a more than 70% stenosis rate in these patients. The stents were implemented in all 42 patients; 21 (50%) of these were male, and the mean age of all patients was 52.74 ± 13.02 years. Thirty-five patients (83.33%) had impaired sensory or motor function of the limbs. In the univariate analysis, 11 morphologic or first-order radiomics features and five clinical features were initially identified as potentially associated with short-term favorable outcomes. Logistic multivariate analysis further indicated that shape-flatness (*p* = 0.04, Odd ratio (OR) = 169.02, 95% CI: 1.30–22,026.5) and first-order-minimum (*p* = 0.02, OR = 94.63, 95% CI: 1.93–4592.5) might be independently related to post-stenting outcomes. A prediction model constructed based on the above morphologic and first-order features showed an AUC of 0.82 in this small cohort.

**Conclusion:**

Plaque-based radiomic features, which describe the shape and voxel characteristics extracted from HR-MRI data, are associated with the short-term outcomes of patients treated with stent implementation.

## Abstract (es)

**Objetivo:**

Evaluar la viabilidad de la radiómica basada en placas extraída a partir de imágenes por resonancia magnética de alta resolución (RM-AR) para evaluar los resultados a corto plazo del tratamiento endovascular en pacientes con estenosis sintomática de la arteria intracraneal.

**Métodos:**

Se realizó una RM-AR a pacientes con estenosis sintomática de la arteria intracraneal. Se extrajeron de la RM-AR los datos radiómicos basados en la placa que describen las características morfológicas y el valor de píxel de la imagen. Asimismo, se recopilaron datos demográficos. El resultado favorable a corto plazo se definió como una tasa de estenosis residual postoperatoria <35 % sin complicaciones perioperatorias. Se llevó a cabo un análisis univariante para identificar las características asociadas con resultados favorables. A partir de los resultados de este análisis, se desarrolló un modelo de predicción utilizando regresión logística. El rendimiento de los modelos clínicos y radiómicos se evaluó utilizando la curva característica operativa del receptor (ROC, Receiver Operating Characteristic) y el área bajo la curva (ABC).

**Resultados:**

Entre Enero de 2022 y diciembre de 2023, se inscribieron 42 pacientes consecutivos con estenosis sintomática de la arteria intracraneal. La angiografía por sustracción digital (DSA, digital subtraction angiography) reveló una tasa de estenosis superior al 70 % en estos pacientes. A los 42 pacientes se les implantaron stents. Había 21 pacientes varones (el 50 %) y la edad media de todos los pacientes era de 52,74 ± 13,02. Treinta y cinco pacientes (83,33 %) presentaban alteraciones de la función sensorial o motora de las extremidades. En el análisis univariante, se identificaron inicialmente 11 características morfológicas o radiómicas de primer orden y 5 características clínicas como potencialmente asociadas a resultados favorables a corto plazo. El análisis logístico multivariante indicó además que la planitud de la forma (*p* = 0,04, OR = 169,02, IC del 95 %: 1,30–22.026,5) y el mínimo de primer orden (*p* = 0,02, OR = 94,63, IC del 95 %: 1,93–4592,5) podrían estar relacionados de forma independiente con los resultados posteriores a la implantación del stent. Un modelo de predicción creado a partir de las características morfológicas y de primer orden mencionadas anteriormente mostró un ABC de 0,82 en esta pequeña cohorte.

**Conclusión:**

Las características radiómicas basadas en la placa, que describen las características de la forma y los vóxeles extraídas de la RM-AR, están asociadas con los resultados a corto plazo de los pacientes tratados con la implantación de stents.

## 1. Introduction

Stroke is the second leading cause of death globally and in China, respectively [1, 2]. Furthermore, intracranial atherosclerotic stenosis (ICAS) is the most common cause of stroke in Asia (accounting for 33–67% of previously reported cases) [3, 4]. While the materials and techniques for cerebrovascular treatment continue to develop and advance, there remain significant differences in the formulation of individualized plans and clinical implementation regarding interventional treatment for symptomatic intracranial arterial stenosis. Therefore, identifying patients eligible for angioplasty and stent implantation pre-treatment would be imperative for improved outcomes.

Several prospective studies—including the Stenting of Symptomatic Atherosclerotic Lesions in the Vertebral or Intracranial Arteries (SSYLVIA), The Stenting and Aggressive Medical management for the Preventing Recurrent Stroke in Intracranial Stenosis (SAMMPRIS), and Effect of a Balloon-Expandable Intracranial Stent vs Medical Therapy on Risk of Stroke in Patients With Symptomatic Intracranial Stenosis (VISSIT) studies—reported an incidence of stroke complications of ⁓30% after endovascular treatment [5, 6, 7, 8], significantly higher than that in the conservative treatment group. In this regard, it is noteworthy that with improved materials, the efficacy of angioplasty and stent implantation may not be inferior to medical therapy [9]. In other words, some patients with intracranial artery stenosis could still benefit from endovascular treatment.

High-resolution magnetic resonance imaging (HR-MRI) enables thorough, non-invasive assessment of intracranial arterial plaques, even in cases of mild stenosis. Furthermore, HR-MRI-based plaque characteristics could predict the presence of high-risk intracranial plaques [10, 11]. Nonetheless, HR-MRI images contain numerous quantifiable features that could further influence plaque characterization and treatment procedures, highlighting the need for advanced data extraction methods.

Radiomics, a computer-assisted, high-throughput method for extracting quantifiable data from medical images, could identify imaging features that are not visible to the naked eye [12, 13]. Some of its benefits include non-invasiveness, quantifiability, and accuracy. Radiomics has recently gained more popularity in the diagnosis and prognostic evaluation of vascular diseases [14, 15, 16, 17, 18]. However, its application in intracranial arterial plaques remains limited. Particularly, there are limited patient selection procedures that could be employed to determine the plaques most likely to improve after stenting without complications from endovascular treatment.

Besides reducing the postoperative stroke rate, appropriate patient selection could improve the safety of angioplasty and stent implantation, highlighting the need for such pertinent research, which remains rare. This retrospective study reviewed patients with intracranial artery stenosis who were treated with angioplasty and stent implantation at our hospital. All patients underwent HR-MRI before treatment. Perioperative stroke events and residual postoperative stenosis immediately after the procedure were recorded. Additionally, the plaques’ morphological features and descriptive voxel characteristics were examined with HR-MRI using radiomics to assess their impact on short-term outcomes, facilitating the identification of patients suitable for endovascular treatment.

## 2. Methods

### 2.1 Patient Selection

Symptomatic ICAS patients with a stenosis rate of 70–99% confirmed via digital subtraction angiography (DSA) were retrospectively reviewed in our department between January 2022 and December 2023. Although regular conservative medication was administered to all candidates for interventional treatment initially, some still experienced recurrent ischemic stroke (IS) or transient ischemic attack (TIA). Additionally, the interventional procedure was also performed on patients whose families and themselves strongly requested it to avoid subsequent ischemic events. Notably, angioplasty and stenting should be performed ≥1 month after the last IS event has stabilized. Consequently, all patients underwent HR-MRI before treatment. After a thorough evaluation, a chief neurosurgeon with >10 years of surgical experience performed the procedure. The inclusion criteria were: (1) Patients aged 18–80 years; (2) ICAS patients; and (3) Patients with complete HR-MRI data before stent implementation. On the other hand, the exclusion criteria were: (1) Patients with acute stroke; and (2) Patients without, or who refused to provide clinical data.

### 2.2 Demographic and Clinical Data Collection

Demographic information gathered encompassed patients’ age, gender, and medical history. The clinical symptoms monitored included muscle weakness, sensory disturbances, aphasia (language impairment), headaches, and hypopsia (reduced visual acuity). Additionally, radiographic data were collected, with specific measurements as follows: Vessel diameter at the stenosis location, proximal vessel diameter (defined as the diameter of the vessel segment 1 cm upstream from the stenosis), distal vessel diameter (defined as the diameter of the vessel segment 1 cm downstream from the stenosis), mean vessel diameter (the average diameter of the distal, proximal, and middle segments of the target vessel), preoperative stenosis rate (computed using the formula: 1 minus [vessel diameter at the stenosis ÷ mean vessel diameter]), postoperative residual stenosis rate and stenosis improvement rate (computed as the difference between the preoperative stenosis rate and the postoperative residual stenosis rate). Two experienced neurosurgeons, each with five years of neuroradiology experience, manually took measurements from diagnostic DSA images. Mean values from both neurosurgeons represented the final measurements, providing a comprehensive overview of the vascular condition and treatment outcomes. The stenosis measurements adhered to the WASID criteria (established by The Warfarin-Aspirin Symptomatic Intracranial Disease Study, using the formula: 1 – [Ds / Dn] × 100 (The Ds represents the most severe diameter stenosis and the Dn represents the diameter of the reference normal vessel, which at the widest, parallel, non-tortuous segment)). We also collected preoperative and immediate postoperative modified rankin scale (mRS) and national institutes of health stroke scale (NIHSS) scores. Furthermore, based on postoperative computed tomography (CT) scans and patient symptoms, stroke complications were recorded.

### 2.3 HR-MRI Original Data Collection

Before treatment, the patients underwent HR-MRI, performed using a 3-T MRI scanner (Skyra; Siemens Healthcare, Erlangen, Germany), fitted with a 20-channel phased array head and neck coil, with two-dimensional (2D) high-resolution black blood T1- and T2-weighted fast-spin-echo sequences acquired post-scanning. The T1 images were acquired both pre- and post-contrast (gadolinium). After the initial multi-plane sequence, the patients underwent axial 3D time-of-flight (TOF) MR angiography to identify the location of the stenosis. Subsequently, HR-MRI scanning was performed in planes perpendicular to the artery’s longitudinal orientation. The scan parameters were: Both T1- and T2-weighted sequences acquired with 12 × 2-mm-thick slices; Field of View: 100 mm × 100 mm; matrix: 256 × 320; and in-plane resolution: 0.4 mm × 0.3 mm, the true spatial resolution could reach 0.5 mm. Individually, T1 images had repetition time (TR)/echo time (TE) of 581 ms/18 ms, echo train length (ETL) of 4, and number of excitations (NEX) of 4, while T2 images had TR/TE of 2890 ms/46 ms; ETL of 20; and NEX of 3.

### 2.4 Endovascular Procedure 

All procedures were performed under general anesthesia. After puncturing the femoral artery using the Seldinger technique, the 6-F guide catheters, along with an 8-F short sheath, were routinely placed. Sometimes, a 6-F long sheath and an intermediate catheter were needed for some issues. Under roadmap guidance, a microguidewire [Synchro-2 (0.014″ × 200 cm, Stryker, Kalamazoo, MI, USA) or ASHKI (0.014″ × 200 cm, ASAHI INTECC CO., LTD., Nagoya, Aichi Prefecture, Japan) 0.014 inch (0.356 mm)] of 2/3 m in length was selected based on the balloon after identifying the true lumen. Intracranial expansion balloons [Gateway (Boston, MA, USA), Monorail (Boston, MA, USA), Xinwei (Xinwei Medical Technology Co., Ltd., Shanghai, China), and Sino (2.5–5.0 mm × 12–25 mm, Sino Shenchang Medical Technology Co., Ltd., Tianjin, China)] were used to expand the lesion. Subsequently, the attending surgeon released a self-expanding stent (Wingspan, Stryker, Kalamazoo, MI, USA; Enterprise, Codman Neuro, Raynham, MA, USA; Neuroform-EZ, Stryker, Kalamazoo, MI, USA; Solitaire, Medtronic, Minneapolis, MN, USA). All measurements were taken at the same site pre- and post-treatment. Postoperative Dyna CT scans were routinely performed to check for intracranial hemorrhage.

### 2.5 Plaque-based Radiomics Features Extraction 

**Fig. 1.**
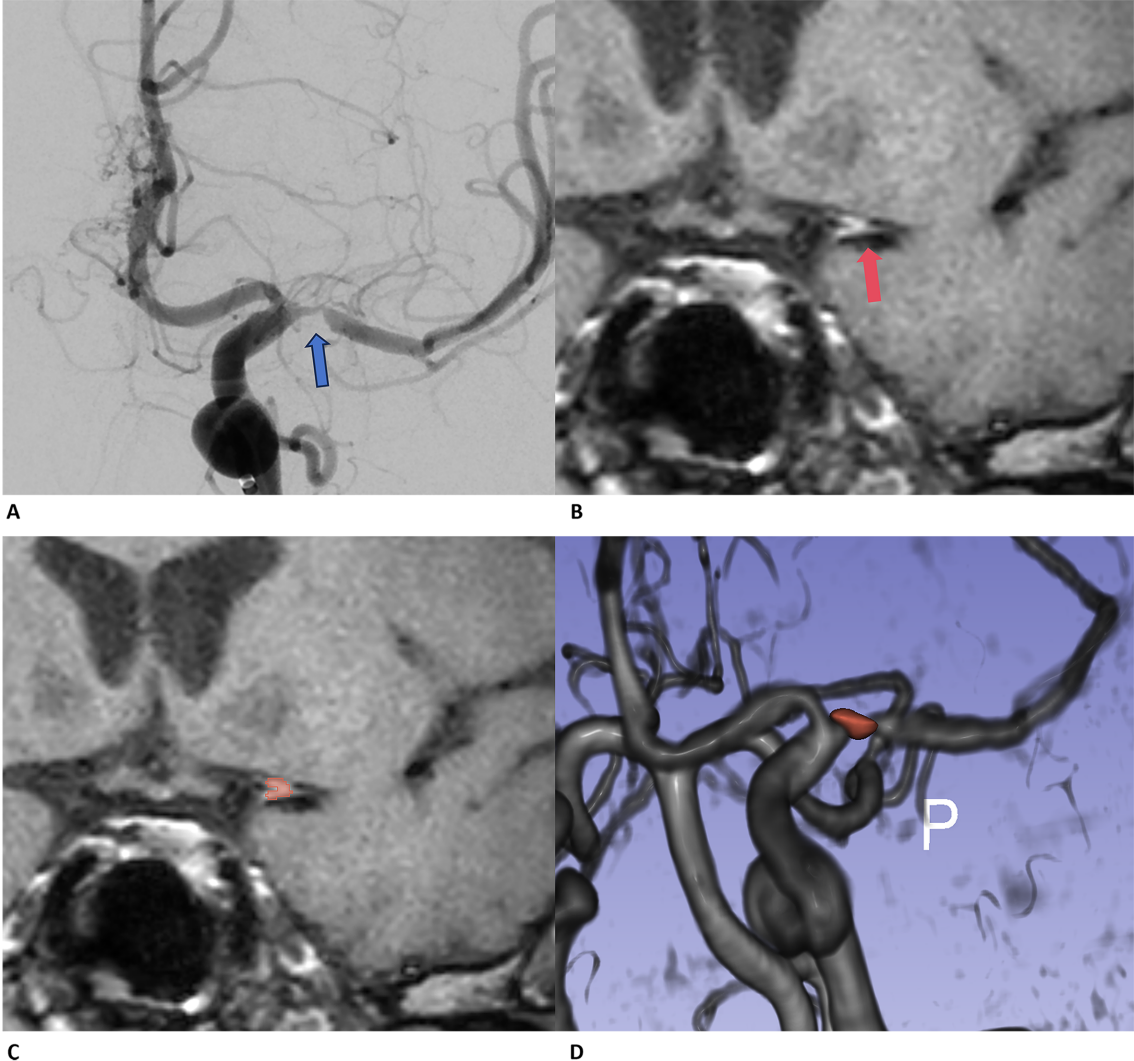
**The illustration of plaque segments based on high-resolution MRI images**. (A) The preoperative angiography indicates the location of the stenosis site, revealing that this patient has nearly 95% stenosis (blue arrow); (B) and (C) show the segmentations of plaques on Black-Blood-MSDE images (red arrow and red area). (D) The reconstruction of the 3D time-of-flight (3D-TOF) series clearly shows the accurate segmentation of plaque areas. MRI, magnetic resonance imaging; MSDE, motion sensitized driven equilibrium.

The plaque was delineated manually using the open-source software 3D-Slicer (version 4.10.2, Massachusetts Institute of Technology, Cambridge, MA, USA) [19]. Images were downloaded in digital imaging and communications in medicine (DICOM) format and read with a DICOM viewer built into 3D-Slicer. Subsequently, the segment editor module was used to delineate the plaque boundary at each level on T1-Black Blood Motion Sensitized Driven Equilibrium (MSDE) images. The plaque exhibited a slightly higher signal in the HR-MRI T1 black blood sequence, clearly distinguishing the locations of the lumen and plaque. Meanwhile, the 3D modeling of the patients’ cerebrovascular was reconstructed in advance with the segmentation module based on the patients’ 3D-TOF sequence, clearly distinguishing the stenosis and plaque locations. Two neuroradiologists (Kai Mao and Lingyou Chen) segmented the plaques independently, with reference to DSA and 3D modeling (Fig. 1) [20]. The morphological and first-order features describing the plaques were then extracted using Pyradiomics (version 2.1, developed and maintained by the Radiomics Community, including collaborators from the Netherlands Cancer Institute - Antoni van Leeuwenhoek and Harvard Medical School, Boston, MA, USA; website: https://pyradiomics.readthedocs.io/) in Python, including intensity (maximum intensity, average intensity, and standard deviation of intensity, among others) and shape-based (area, length, and so on) features [21, 22].

The groups were compared based on significant radiomics features selected using univariate analysis. A residual stenosis rate <35% post-treatment (the quartile of postoperative residual stenosis) with no perioperative complications indicated a favorable prognosis. Independent factors affecting a favorable prognosis were identified using a logistic regression model.

### 2.6 Statistical Analysis

Continuous variables were presented as medians [Interquartile Ranges (IQRs)] or mean ± Standard Deviation (SD), and the differences between different prognosis groups were compared by Student’s *t*-test if the variables had equal variance. The Mann-Whitney U test was used if the variables had unequal variance. Categorical Variables were expressed as counts (N) and percentages (%), with the chi-square test or Fisher’s exact test used for inter-group comparisons. Significant features between the two groups were identified using univariate logistic regression, with a *p* < 0.2 threshold employed to screen features for multivariate logistic regression. After conducting univariate analysis, we performed data normalization on the remaining radiomic features to prevent excessively large differences in statistical values. On the other hand, multivariate analysis was used to identify independent factors related to treatment outcomes, with a *p* < 0.05 cutoff used to select prognosis-associated factors. Significantly different clinical and radiomics features in the multivariate analysis were used to develop predictive models via logistic regression. The models’ discrimination ability was assessed using Receiver Operating Characteristic (ROC) curves, which were compared using DeLong tests. All statistical analyses were performed using R (v3.4.0, R Foundation for Statistical Computing, Vienna, Austria) and SPSS (v24.0, IBM Corp., Armonk, NY, USA) software.

## 3. Results

### 3.1 Demographic Data

From February 2022 to January 2023, a total of 98 patients with intracranial atherosclerotic stenosis (ICAS) underwent endovascular intervention at our hospital. Among these patients, 23 were excluded from the study for failing to complete preoperative HR-MRI; 12 were excluded because they strongly requested surgery without rigorous conservative medical treatment; and an additional 21 were excluded due to incomplete clinical or imaging data. Eventually, 42 consecutive patients with symptomatic intracranial artery stenosis were enrolled. The mean age of patients was 52.74 ± 13.02 years, including 21 (50.00%) male patients. The proportions of patients with hypertension (HTN) and diabetes were 25 (59.52%) and 12 (28.57%), respectively. Furthermore, 35 patients (83.33%) had motor/sensory impairments in their limbs, 16 (38.10%) suffered from aphasia, and 11 (26.19%) had symptoms that presented only as dizziness and headache. Additionally, the median preoperative NIHSS and mRS scores were 1.00 (0, 2) and 1.00 (0, 1), respectively. Table 1 details the patients’ demographic data.

**Table 1.** **Demographic data of enrolled patients**.

Variables		Value
Gender (n, %)		
	Female	21 (50.00)
	Male	21 (50.00)
Age (y, mean ± SD)		52.74 ± 13.02
Clinical symptoms		
	Dizziness and headache (n, %)	11 (26.19)
	Aphasia (n, %)	16 (38.10)
	Limb weakness or numbness (n, %)	35 (83.33)
Past medical history (n, %)		
	Diabetes	12 (28.57)
	Hypertension	25 (59.52)
Preoperative mRS (median with interquartile range)		1.00 (0.00, 1.00)
The vessel diameter of the stenosis site (mm, mean ± SD)		0.81 ± 0.34
Proximal vessel diameter of stenosis (mm, mean ± SD)		3.84 ± 1.09
Distal vessel diameter of stenosis (mm, mean ± SD)		3.12 ± 0.90
The mean vessel diameter (mm, mean ± SD)		3.45 ± 0.89
Preoperative stenosis rate (%, mean ± SD)		77.0 ± 6.0
Residual stenosis rate (%, median with interquartile range)		24 (12, 35)
24 h mRS after procedure (median with interquartile range)		0 (0, 1)
mRS before discharge (median with interquartile range)		0 (0, 1)

mRS, modified rankin scale.

### 3.2 Radiographic Data 

All arteries involved were part of the anterior circulation. The left Middle Cerebral Artery, left Internal Carotid Artery (ICA), right MCA, and right ICA were affected in 24 (57.14%), 3 (7.14%), 10 (23.81%), and 5 (11.90%) cases, respectively. During endovascular treatment, one patient (2.38%) with severe stenosis of the right ICA (C6-C7 segment) ruptured the right posterior communicating artery aneurysm. In pre-operative radiographic data, the mean vessel diameter at the site of stenosis was 0.81 ± 0.34 mm. Additionally, the mean proximal and distal vessel diameters were 3.84 ± 1.09 mm and 3.12 ± 0.90 mm, respectively. Furthermore, the overall mean vessel diameter was 3.45 ± 0.89 mm, and the mean preoperative stenosis rate was 0.77 ± 0.06 mm. Two patients (4.76%) experienced permanent complications after the procedure. One patient (2.38%) who had a combined posterior communicating aneurysm suffered an intraoperative hemorrhage due to aneurysm rupture and was later discharged, albeit in poor condition with an mRS score of 5. The other patient developed cerebral hemorrhage due to postoperative perfusion pressure breakthrough, with an mRS score of 5 at discharge. All patients’ median postoperative mRS score was 0 (0, 1), with a similar score at discharge. Additionally, the mean improvement in radiographic stenosis post-treatment was 0.54 ± 0.16, and the median postoperative residual stenosis rate was 0.24 (0.12, 0.35). Postoperative residual stenosis <35% and absence of complications indicated a favorable outcome, which was reported in 29/42 patients (69.05%).

### 3.3 Factors Associated With Treatment Outcome

**Fig. 2.**
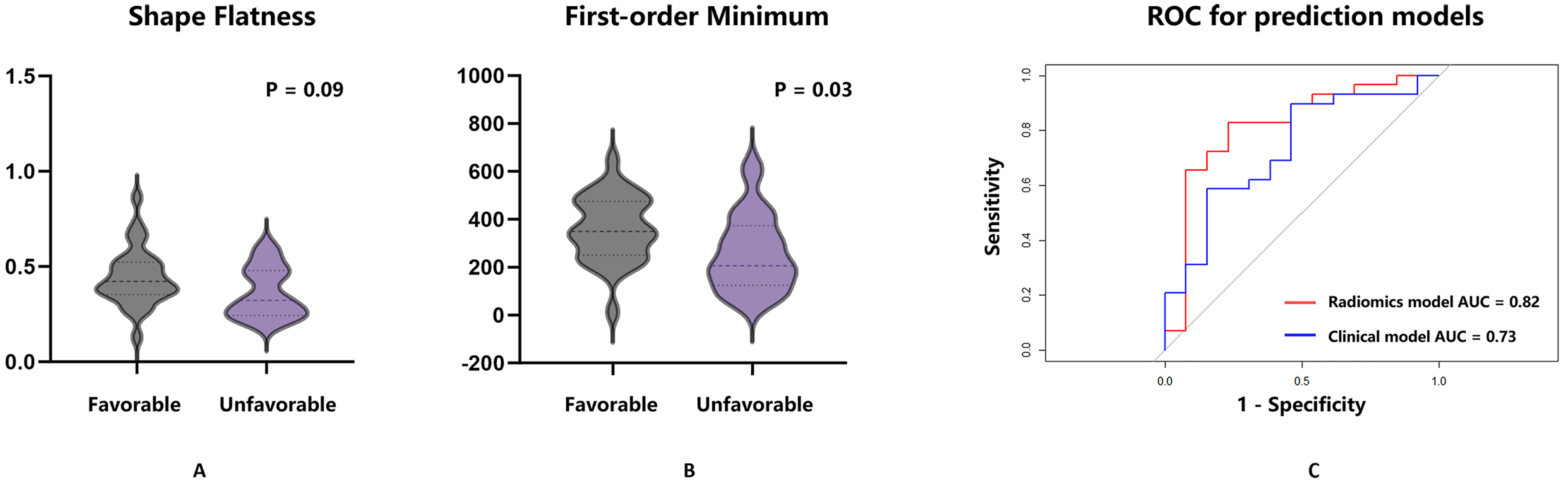
**Shape-flatness and first-order-minimum were the independent factors associated with the favorable outcome**. (A) The difference in shape-flatness between the favorable and unfavorable outcome groups was examined. Univariate analysis showed a *p*-value of 0.09 between the two groups, with the value in the favorable outcome group being higher than that in the unfavorable group (0.44 vs 0.36, *p* = 0.09). (B) The difference in the first-order-minimum between the favorable and unfavorable outcome groups was examined. Univariate analysis showed a *p*-value of 0.03 between the two groups, with the value in the favorable outcome group being higher than that in the unfavorable group (361.00 vs 258.50, *p* = 0.03). (C) The ROC curve for the radiomics prediction model, which used shape-flatness and first-order minimum features with a logistic regression algorithm, is shown in red. The clinical model, based on age and pre-treatment stenosis rate, is in blue. The AUC for the radiomics model is 0.82, while the clinical model is 0.73. The Delong test revealed no significant difference between the two models, and the clinical factors showed no statistical significance in multivariate analysis (*p* > 0.05). ROC, receiver operating characteristic; AUC, area under the curve.

Herein, HR-MRI was used to extract features that quantified shape and described the voxels, including 14 shape-relevant and 19 first-order radiomics features. Univariate logistic regression analysis identified 11 radiomics features as relevant to favorable outcomes, of which five clinical variables correlated with a favorable outcome, including age, vessel diameter at the stenosis site, preoperative stenosis rate, distal vessel diameter, and mean vessel diameter. Table 2 shows the differences between the groups for each feature. Multivariate analysis was performed using binary logistic regression with the forward selection method. Shape-flatness and first-order-minimum were the independent factors that correlated with a favorable outcome (shape-flatness (*p* = 0.04, OR = 169.02, 95% CI: 1.30–22,026.5); first-order-minimum (*p* = 0.02, OR = 94.63, 95% CI: 1.93–4592.5)), with higher mean values in the favorable outcome group than in the unfavorable prognosis group (mean shape-flatness—favorable outcome vs unfavorable outcome: 0.44 vs 0.36, *p* = 0.09; mean first-order-minimum—favorable outcome vs unfavorable outcome: 361.00 vs 258.50, *p* = 0.04). Multivariate analysis further revealed that plaque morphology and first-order features significantly influenced the outcomes. Through logistic regression, these two features were used to construct a prediction model, which had an Area Under the Receiver Operating Characteristic Curve (AUROC) of 0.82 (Fig. 2).

**Table 2.** **The difference between the two groups**.

Variables (mean ± SD)	Favorable outcome (n = 29)	Unfavorable outcome (n = 13)	*p* value	Multivariate odds ratio	*p* value
Age (y)	50.45 ± 13.08	57.85 ± 11.80	0.09*		
Diameter of stenosis (mm)	0.74 ± 0.31	0.97 ± 0.36	0.06*		
Distal diameter of stenosis (mm)	2.97 ± 0.84	3.47 ± 0.96	0.10*		
Mean diameter of stenotic artery (mm)	3.02 ± 0.91	3.30 ± 0.98	0.19*		
Stenosis percentage (%)	0.78 ± 0.07	0.74 ± 0.05	0.09*		
original_shape_Flatness	0.44 ± 0.15	0.36 ± 0.13	0.09*	169.02	0.04**
original_shape_MajorAxisLength	6.96 ± 2.74	8.47 ± 3.44	0.15*		
original_shape_Maximum2DDiameterColumn	7.01 ± 2.13	8.10 ± 2.57	0.16*		
original_shape_Maximum3DDiameter	7.86 ± 2.24	9.21 ± 3.12	0.13*		
original_shape_MeshVolume	32.97 ± 21.85	44.30 ± 23.83	0.15*		
original_shape_SurfaceArea	81.56 ± 38.05	100.47 ± 43.77	0.17*		
original_shape_SurfaceVolumeRatio	2.67 ± 0.52	2.42 ± 0.40	0.11*		
original_shape_VoxelVolume	34.75 ± 22.19	46.31 ± 24.35	0.15*		
original_firstorder_10Percentile	714.87 ± 156.01	614.51 ± 244.96	0.12*		
original_firstorder_Minimum	360.96 ± 136.11	258.52 ± 158.68	0.04**	94.63	0.02**
original_firstorder_Variance	96,267.56 ± 69,975.74	146,648.3 ± 158,848.07	0.18*		

*. Univariate logistic regression identified significant features between the two groups, using a *p*-value threshold of <0.2 for multivariate logistic regression selection. **. *p* value < 0.05.

Univariate analysis identified five clinical factors that influenced a favorable outcome, which were further analyzed separately using multivariate analysis, revealing that age and the preoperative stenosis rate were the independent factors affecting prognosis after endovascular treatment. However, neither of these factors showed a statistically significant difference in multivariate analysis using logistic regression (age, *p* = 0.066; preoperative stenosis rate, *p* = 0.066). Based on the two clinical and radiographic features, a logistic regression model was developed, with an AUROC prediction efficiency of 0.73. DeLong tests revealed no statistical significance between the Radiomics and clinical prediction models (*p* = 0.411, Fig. 2).

## 4. Discussion

**Fig. 3.**
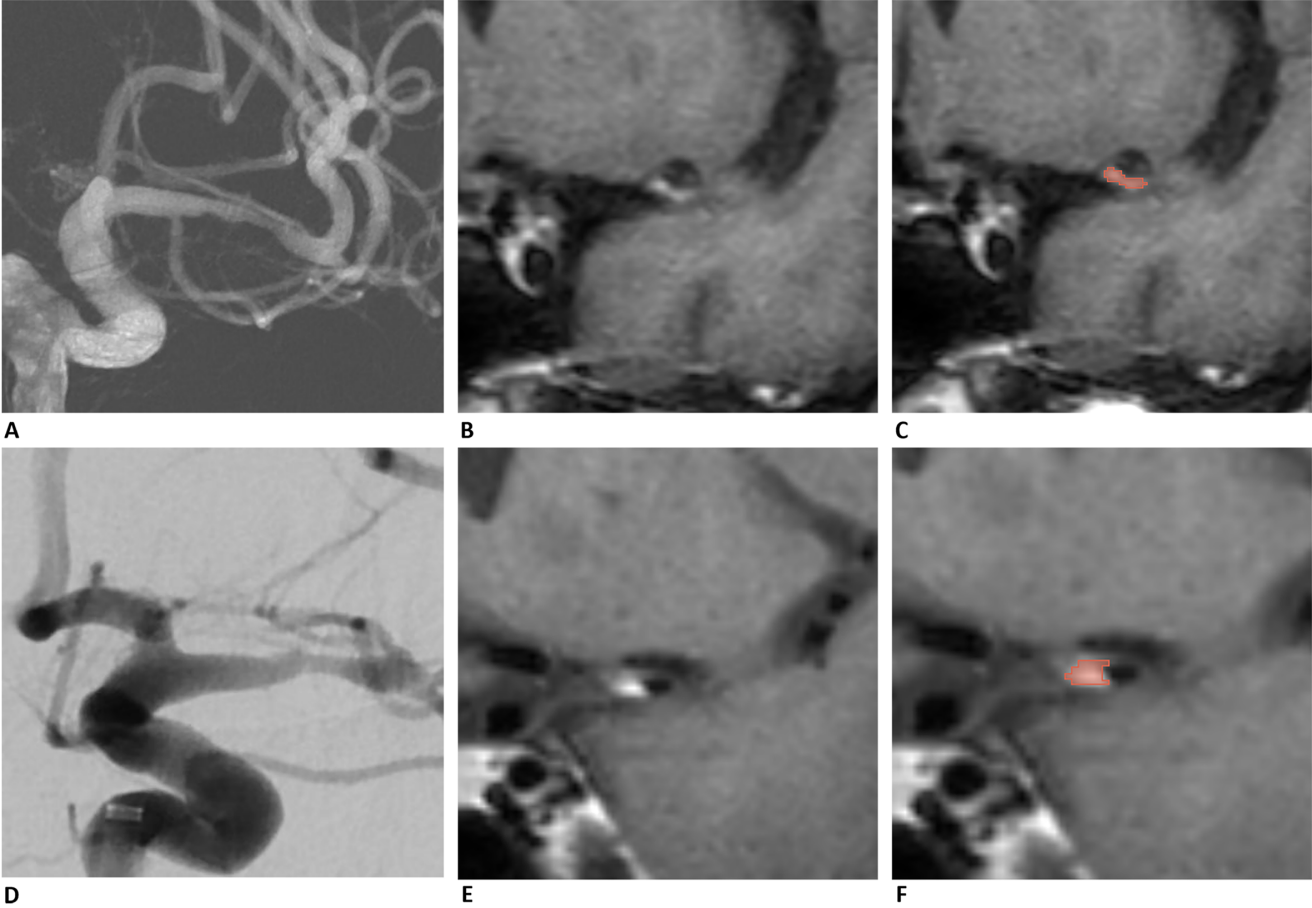
**Two patients with different flatness values are presented in (A–C) and (D–F)**. Patient 1 (A–C) has a flatness value of 0.23, indicating that the plaque shape is relatively flat. In contrast, Patient 2 has a flatness value of 0.83, suggesting a more spherical plaque shape. Images (A) and (D) show the preoperative angiography of the stenosis site, confirming the location of the stenosis. Images (B) and (E) display plaques identified in HR-MRI, which appear as high signals in the black-blood sequence. Images (C) and (F) present the segmentations of the plaques from the two patients, highlighted in red. HR-MRI, high-resolution magnetic resonance imaging.

We examined the impact of various plaque characteristics on stenting using HR-MRI and plaque-based radiomics [10, 23, 24, 25], with shape characteristics and first-order features emerging as independent predictors of outcomes in angioplasty- and stenting-treated ICAS patients. Traditionally, plaque stability correlates with its enhancement, a characteristic of atherosclerotic instability or vulnerability that may indicate plaque progression. We also found that first-order features correlated with plaque enhancement. First-order feature statistics often describe the distribution of voxel intensities within the plaque-defined image area, with the first-order minimum indicating the lowest voxel value within the plaque segmentation in the HR-MRI black blood sequence. Furthermore, plaque stability is conventionally linked to plaque surface irregularity. Irregular and concave plaque surface morphology often implies that the plaque fibrous cap may be eroded, leading to plaque rupture and thromboembolism. Flatness could also preliminarily reflect plaque surface irregularity. Flatness, as a shape value, showed the relationship between the largest and smallest principal components within the ROI, with the values often ranging between 1 (non-flat, sphere-like) and 0 (a flat object or single-slice segmentation). Herein, a higher flatness value and a greater first-order minimum value are both associated with a better prognosis. In other words, the plaque tended to be spherical, and a higher minimum voxel value within the plaque correlated with a better improvement in stenosis post-treatment. Additionally, patients were less likely to experience perioperative complications. Fig. 3 shows two patients with different flatness values. Patient 1 has a flatness value of 0.23, indicating a relatively flat plaque shape, and a postoperative residual stenosis rate of 36%. Conversely, Patient 2 has a flatness value of 0.83, indicating a more spherical plaque shape, with a residual stenosis rate of 10%. Spherical plaques are often distributed around vessel walls, while flatter plaques are generally more laterally arranged, leading to varying treatment outcomes. Compared to Patient 1 (272.72), Patient 2 had a higher first-order minimum (417.99), implying that the plaque characteristics could affect endovascular treatment outcomes based on natural components, especially the morphological and first-order features that describe the plaques’ basic composition [26]. It is also noteworthy that flatness is an important factor describing the ROI. In two previous studies on the use of radiomics + machine learning algorithms to predict intracranial aneurysm stability, flatness emerged as the most critical morphological feature for this prediction [27, 28]. A previous study [29] investigating the use of radiomic features to identify plaque composition in acute stroke patients after thrombectomy has demonstrated that the first-order-median played a critical role in constructing predictive models for plaque composition. Notably, different compositions are associated with variations in thrombectomy duration, number of thrombectomy attempts, and ultimately, patient prognostic outcomes. Consequently, this finding provides a theoretical basis for exploring the association between the first-order-minimum and surgical outcomes after stent placement in the present study. However, we also found that the 95% confidence intervals (95% CIs) of the two radiomic features exhibit considerable width, which may be limited by the small sample size of this study. Thus, this study is merely exploratory research, and additional studies with larger sample sizes are required to further verify their stability.

This study aimed to establish the link between preoperative non-invasive examinations and favorable outcomes following the endovascular treatment of symptomatic ICAS patients. We selected the most suitable patients beforehand, allowing us to effectively improve the procedure’s success rate, reduce associated risks, and enhance patient satisfaction. Previous research on HR-MRI-based plaque radiomics involved the use of ML methods to identify high-risk unstable plaques in ICAS patients [11]. Traditional clinical features such as plaque length, burden, and enhancement emerged as independent predictors of plaque stability, with the conventional model also demonstrating satisfactory area under the curve (AUC) performance. The ML+radiomics features approach further enhanced the model’s accuracy and strength. These findings, along with our results, imply that plaque characteristics could predict both the prognosis and natural history of disease development. In a previous study involving ⁓90 ICAS patients who underwent HR-MRI, prediction models were created using radiomics features of traditional plaque characteristics, identifying vulnerable carotid plaques [10]. These findings suggest that HR-MRI-based radiomics models could more accurately detect vulnerable plaques. However, the combined model and the radiomics alone model showed no significant difference. Moreover, the radiomics models showed consistently higher AUC values for both the training and testing sets, aligning with our findings. Although this study’s clinical features were not significant, traditional clinical and radiographic characteristics should not be overlooked when studying plaques.

Currently, there is no consensus on which ICAS patients should be enrolled for endovascular treatment. Furthermore, several prospective multicentre Randomized Controlled Trials (RCTs) do not recommend endovascular interventional therapy for intracranial stenosis. For instance, in 2011, SAMMPRIS, a multicenter RCT involving 451 patients, reported that the 30-day stroke or death rate was higher in the stent implantation group (14.7%) than in the conservative treatment group (5.8%). Although SAMMPRIS was the first multicenter RCT in the world, its conclusions raised concerns regarding the effectiveness of endovascular interventions [7]. The VISSIT study, conducted during the same period, reached similar conclusions, with a stent prognosis (stroke or death in the stent group of 24.1%) significantly worse than that of the conservative group (3.1%) post-treatment [8]. Additionally, CASSISS (Effect of Stenting Plus Medical Therapy vs Medical Therapy Alone on Risk of Stroke and Death in Patients With Symptomatic Intracranial Stenosis), a multicenter prospective RCT based on recent advancements in materials and endovascular techniques, revealed that endovascular treatment for select patients was not inferior to conservative medical treatment. This study involved 358 patients and revealed stroke incidences of 8.0% and 7.2% in the stent and medication groups, respectively, with no significant difference between the two groups [9]. It was worth noting that in recent years, a multicenter, prospective, randomized controlled clinical trial from China — the BASIS study (Balloon Angioplasty versus Medical Management for Intracranial Artery Stenosis) — has achieved breakthrough progress. It was the first to confirm that for patients with symptomatic severe intracranial arterial stenosis (sICAS), the efficacy of simple balloon angioplasty combined with medical management is superior to that of medical management alone, providing new evidence for the treatment of this high-risk disease. Therefore, it was necessary to screen for suitable patients to undergo interventional treatment. In our research, the radiographic features describing voxel distribution demonstrated predictive value for a good prognosis among patients with intracranial artery stenosis after endovascular treatment, indicating that plaque characteristics correlated with patients’ short-term prognosis. Previous research on Endovascular Thrombectomy (EVT) for Acute Ischemic Stroke (AIS) also revealed that different plaque components significantly impacted treatment procedures and prognosis [30, 31, 32, 33], implying that patients suitable for endovascular treatment could be identified using plaque-based radiomics. We recorded two major complications after the procedure. One patient suffered a hemorrhage due to the rupture of an aneurysm located at a site different from the stenosis. This phenomenon could be attributed to several factors. First, the stent changes blood flow distribution; after the stent is placed, it might change the hemodynamic state of the aneurysm (e.g., increased shear force flowing into the aneurysm). Second, the stent could increase the perfusion pressure; after blood flow is restored, the pressure in the aneurysm may rise immediately. Third, excessive blood pressure post-treatment could increase the pressure in the aneurysm, inducing rupture. Fourth, the use of anticoagulants or antiplatelet drugs after the procedure may aggravate the risk of rupture. Another patient suffered a cerebral hemorrhage after the procedure, a phenomenon that could also be attributed to several factors, but was different from aneurysm rupture. First, ischemia-reperfusion injury (IRI); blood flow reconstruction may be too fast, and sudden restoration during the procedure may cause a rapid filling of blood vessels beyond the regulatory capacity. Second, the stent’s mechanical effect on the blood vessel wall; stent implantation may cause local vascular endothelial damage or inflammatory response, elevating the risk of perfusion breakthrough. Third, inadequate or excessive BP control after the procedure; HTN may aggravate perfusion breakthrough, and excessive BP reduction may cause re-ischemia. Notably, the patients’ clinical data in this study did not contribute to the final model. Furthermore, neither of the two clinical features was significant in the prediction model built using only the clinical features. Nonetheless, the potential impact of traditional clinical features on patient prognosis should not be overlooked in future studies.

Some limitations of our study are exist. First, due to the sample size limitation, the data were insufficient, and therefore, this study was not compared with various advanced scanning techniques. Second, the patients included in the study were all obtained from a single center, which may contribute to the lack of adequate scalability of the classifier. Third, only anterior circulation stenosis, was enrolled in this study; this may limit the range of application of the research results. Finally, in the univariate analysis, a threshold of *p* < 0.2 was used for variable screening—this threshold is higher than the commonly adopted *p* < 0.1 in univariate analyses for preliminary variable selection. Notably, two variables, shape-flatness and first-order-minimum, demonstrated *p*-values < 0.1 in the univariate analysis and with *p*-values < 0.05 in the multivariate analysis. In future studies, more data from multiple centres will be required to consistently verify the stability of our results. Additionally, follow-up data from patients will be necessary to identify clinical and radiomic features that can predict long-term outcomes for patients.

## 5. Conclusion

The plaque-based radiomic features, which describe the shape and voxel characteristics extracted from HR-MRI, are associated with the short-term outcomes of patients suffering from symptomatic intracranial artery stenosis. These features effectively reflect the characteristics of the plaque and can provide preliminary predictions about the prognosis following interventional treatment.

## Availability of Data and Materials

The original data can be provided by the corresponding author upon reasonable request. The version of the provided original data is completely anonymized.
